# Supplementary material for: Medial prefrontal cortex and anteromedial thalamus interaction regulates goal-directed behavior and dopaminergic neuron activity
Source: Nat Commun. 2022 Mar 16;13:1386. doi: 10.1038/s41467-022-28892-7 (PMC8927595; doi:10.1038/s41467-022-28892-7)
Supplement: Supplementary file 1 — Supplementary Information [file 41467_2022_28892_MOESM1_ESM.pdf]

Supplementary Information for

Medial prefrontal cortex and anteromedial thalamus interaction regulates goal-directed behavior and dopaminergic neuron activity

Chen Yang, Yuzheng Hu, Aleksandr D. Talishinsky, Christian T. Potter, Coleman B. Calva, Leslie A. Ramsey, Andrew J. Kesner, Reuben F. Don, Sue Junn, Aaron Tan, Anne F. Pierce, Céline Nicolas[1], Yosuke Arima, Seung-Chan Lee, Conghui Su, Jensine M. Coudriet, Carlos A. Mejia-Aponte, Dong V. Wang, Hanbing Lu, Yihong Yang, and Satoshi Ikemoto

Correspondence to: satoshi.ikemoto@nih.gov

This PDF file includes:

Supplementary Note 1

Supplementary Figures 1 – 11

Supplementary Tables 1 – 2

Supplementary References

SUPPLEMENTARY NOTE 1

Brain structures are often identified and named without knowing their functions or connectivity. As a result, structural names could cause confusions when subsequent findings begin to suggest their functional roles. One of such structures is the orbital cortex. This label is not based on their function or connectivity, but their location - above the eye sockets, i.e., “orbital”. Therefore, while the medial, ventral, and lateral orbital regions share this label, they may not necessarily be grouped together. Indeed, subsequent connectivity work suggested that the medial and ventral orbital regions belong to the medial division of the PFC, while the lateral and dorsolateral orbital regions belong to the lateral division of the PFC, which also include the anterior insular area [1,2]. The present study denotes the medial and lateral divisions of the PFC as the medial PFC and orbital PFC, respectively. Similarly, the labels: the area 1 (dorsal part) and area 2 (ventral part) of the cingulate cortex in rodents respectively belong to two different divisions: the motor PFC and the medial PFC [1,2].

SUPPLEMENTARY FIGURES 1 – 11

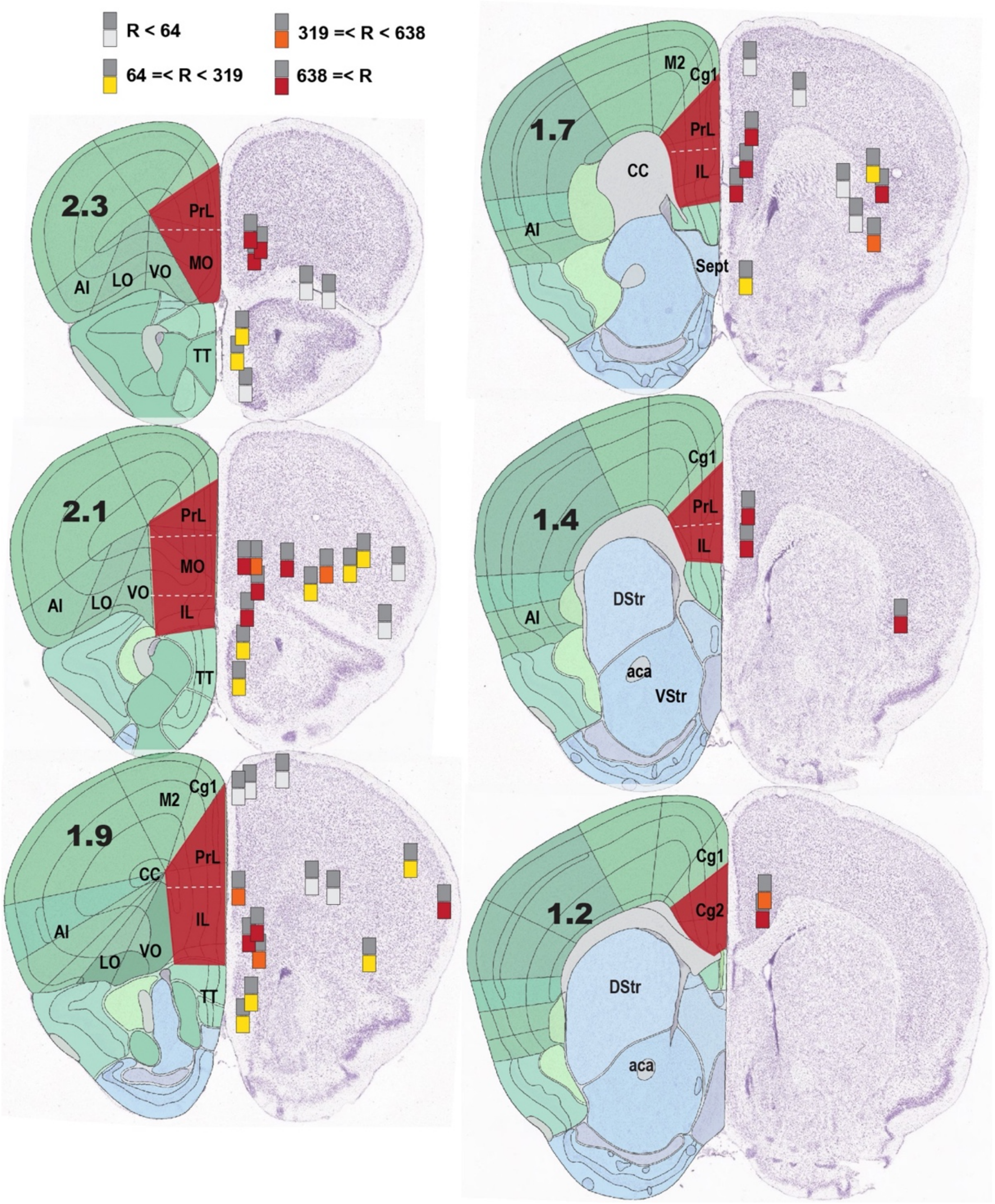

Suppl. Fig. 1. The sites and effectiveness of optogenetic stimulation delivered at the PFC and TT in ICSS. Each dark-gray square indicates the tip of optic fiber, which is accompanied with a colored square below, indicating one of four levels of ICSS rates. Coronal sections were adopted from Allen Mouse Brain Atlas© of Allen Institute for Brain Science available at: https://mouse.brain-map.org/static/atlas. Abbreviations: AI, anterior insular cortex; CC, corpus collosum; Cg1, dorsal anterior cingulate cortex; Cg2, ventral anterior cingulate cortex; IL, infralimbic cortex; LO, lateral orbital cortex; M2, secondary motor cortex; MO, medial orbital cortex; PrL, prelimbic cortex; TT, tenia tecta; VO, ventral orbital cortex. Note that although some rodent brain atlases distinguish the IL from the dorsal peduncular cortex[3,4], others do not[1,2,5], and the present study adopted the latter. Similarly, the AI on the drawing included the anterior insular, dorsal insular, granular insular, and dysgranular insular cortices.

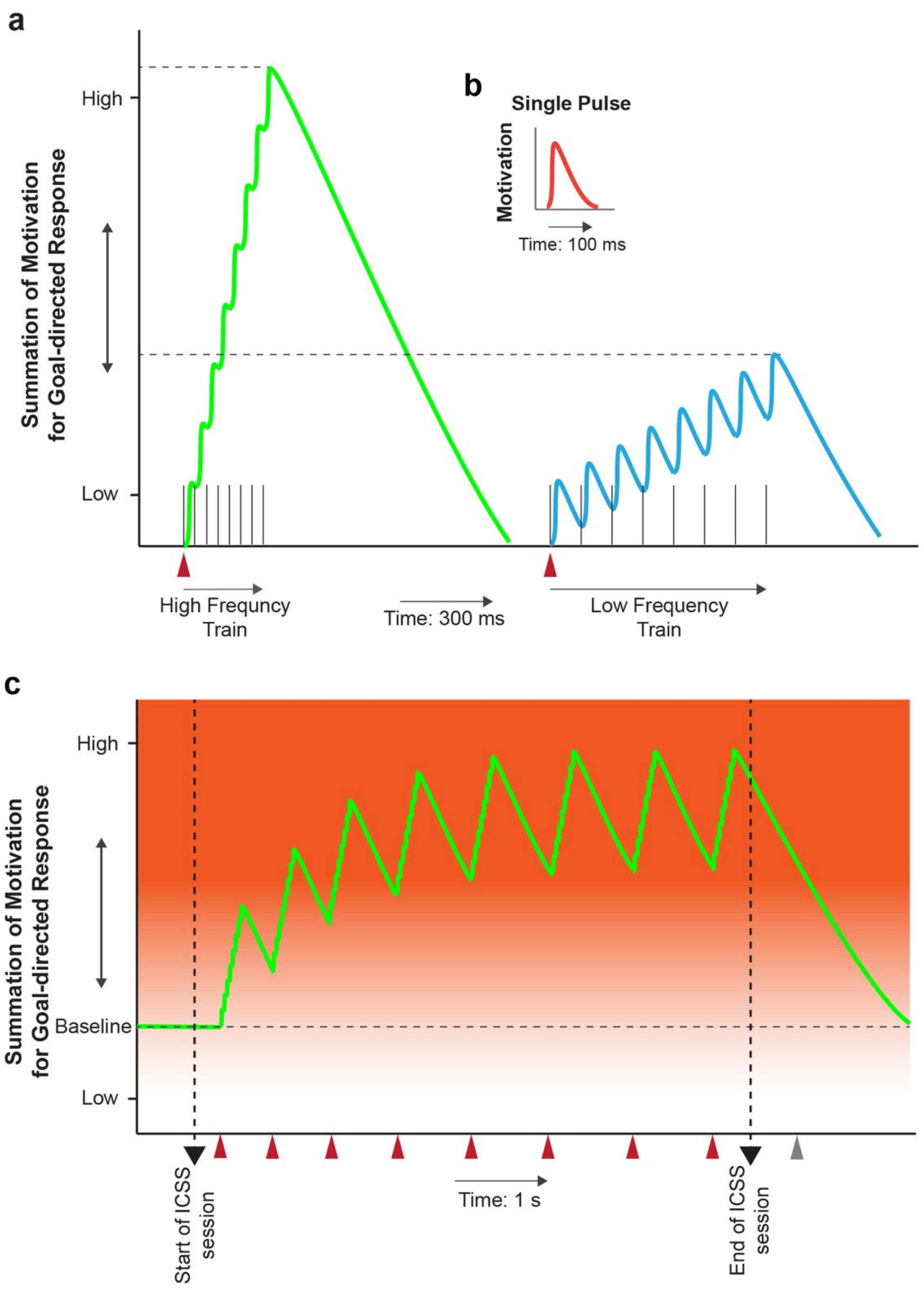

Suppl. Fig. 2. Conceptual models explaining how pulse interval and train interval play important roles in ICSS rates and goal-directed motivation. a Model depicting the summation effects on motivation of a high-frequency and a low-frequency trains of 8 pulses. Each pulse triggers a small motivation effect (b), which decays quickly. When pulses are delivered close in time, they summate to elicit a significant effect on motivation. The arrowhead indicates the onset of a pulse train. **c** Model depicting the summation effect of trains delivered closely in time. When trains occur close in time, the proximity in time allows them to summate, resulting in an increase in motivation for goal-directed behavior, reflected in a high rate of lever responding. The arrowheads with maroon and gray colors indicate the time of lever response reinforced and not reinforced, respectively. Similar models are suggested to explain the relationship between dopamine and ICSS rate[6].

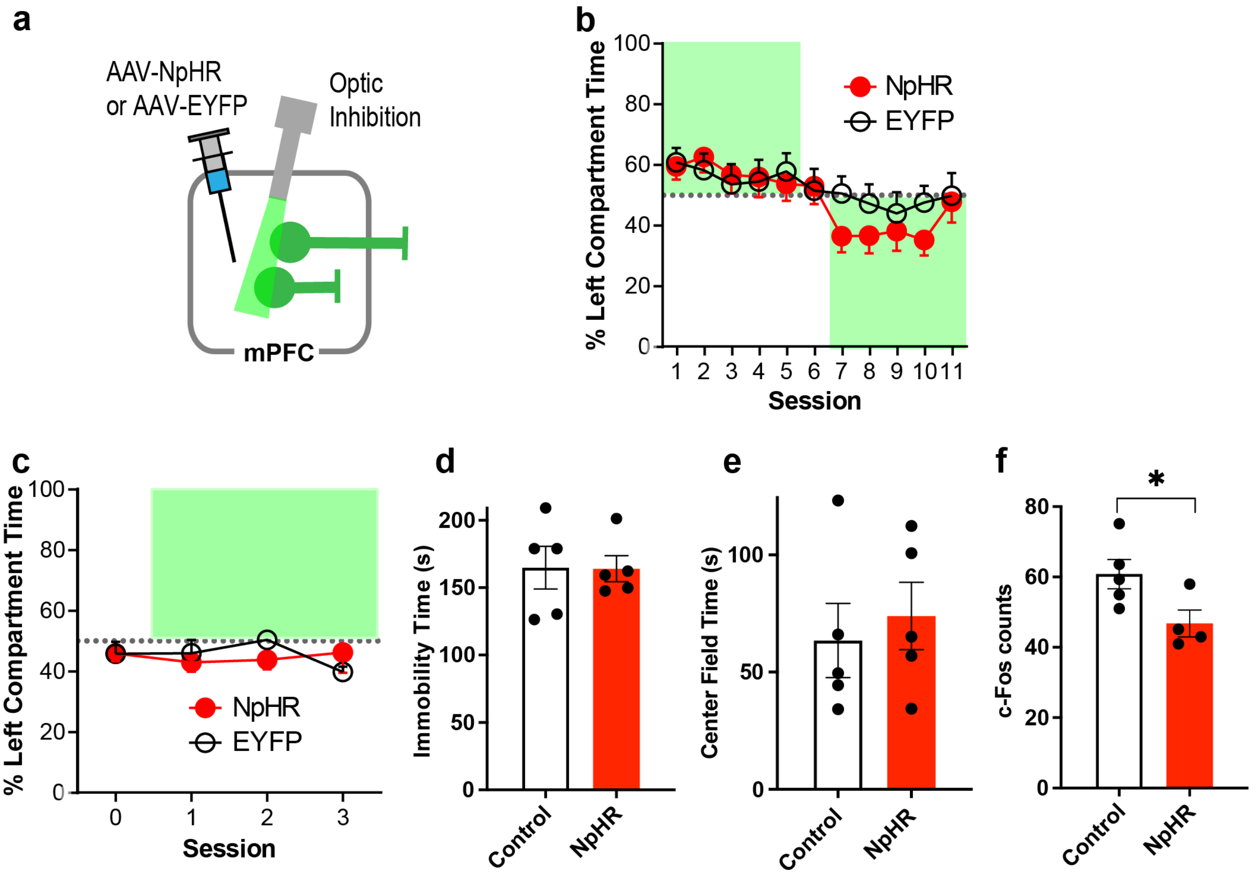

Suppl. Fig. 3. Effects of the inhibition of mPFC neurons on affective state. The results described in this figure suggest that tonic activity of mPFC neurons is not involved in suppressing negative emotional states. **a** Mice received an injection of AAV encoding the gene for halorhodopsin-3.0 (NpHR) or EYFP only and an optic fiber at the mPFC. **b** Real-time place-preference data with unilateral inhibition. NpHR: n = 6; EYFP: n = 6. Data (means ± SEM) are derived by the equation: (left compartment time – right time) / (left time + right time) * 100. Data points fell in green squares indicate relative preference to the stimulation compartment. **c-f** Bilateral inhibition data with two groups NpHR (n = 5) and EYFP (n = 5, except c-Fos counts: n = 4). **c** Real-time place-preference data. Photostimulation was delivered in the left compartment for sessions 1-3, but not session 0. Data (means ± SEM) are derived by the equation: (left compartment time – right time) / (left time + right time) * 100. **d** Mean immobility times (± SEM) during forced swim. **e** Mean center-field times (± SEM) in open field. **f** To confirm that the NpHR manipulation inhibited mPFC neurons, the mice were placed in a novel chamber for 30 min with mPFC photostimulation before being euthanized. mPFC c-Fos expressions were then compared between the experimental and control groups. The data are mean c-Fos counts (± SEM) in the mPFC. The NpHR group had significantly less c-Fos expression than the control group (*$P = 0.0230$, $t_7 = 2.42$; unpaired, one-tailed t-test), suggesting that the photostimulation inhibited mPFC neurons during the tests described above.

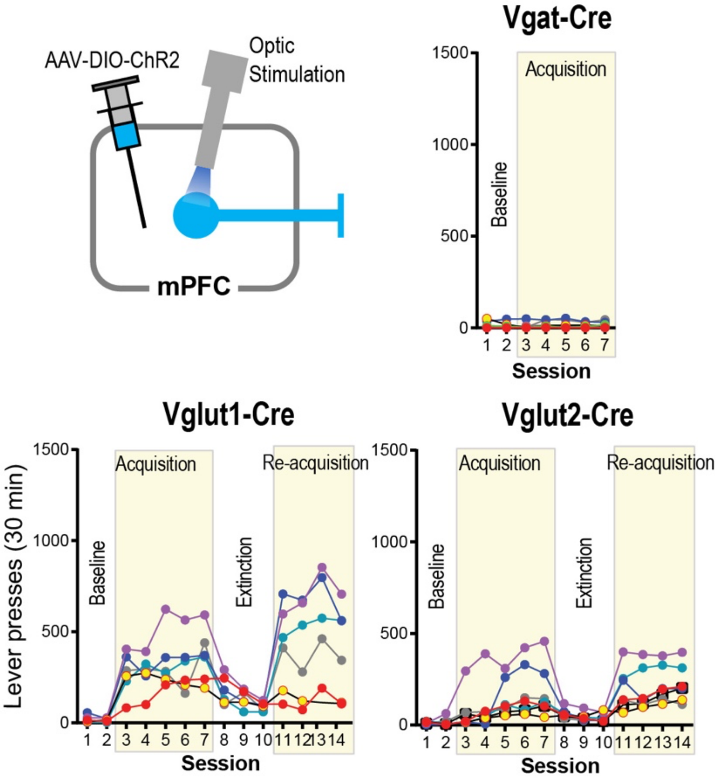

Suppl. Fig. 4. Glutamatergic neurons are important in goal-directed motivation induced by mPFC stimulation. While most cortical glutamate neurons are vesicular glutamate transporter type 1 (Vglut1), the vicinity of the IL contains Vglut2 neurons. Cre-dependent AAV-ChR2-EYFP was injected into the mPFC followed by the implantation of an optic fiber at the same region of transgenic mice: Vlgut1-Cre, Vglut2-Cre, or Vgat-Cre mice. The mice received photostimulation (a train of 8 pulses at 25 Hz) in sessions 3-7 and 11-14, while no photostimulation in sessions 1-2 or 8-10. mPFC photostimulation reinforced responding of all Vglut1-Cre (n = 6) and Vglut2-Cre (n = 6) mice, whereas the same stimulation failed to reinforce responding of Vgat-Cre mice (n = 6).

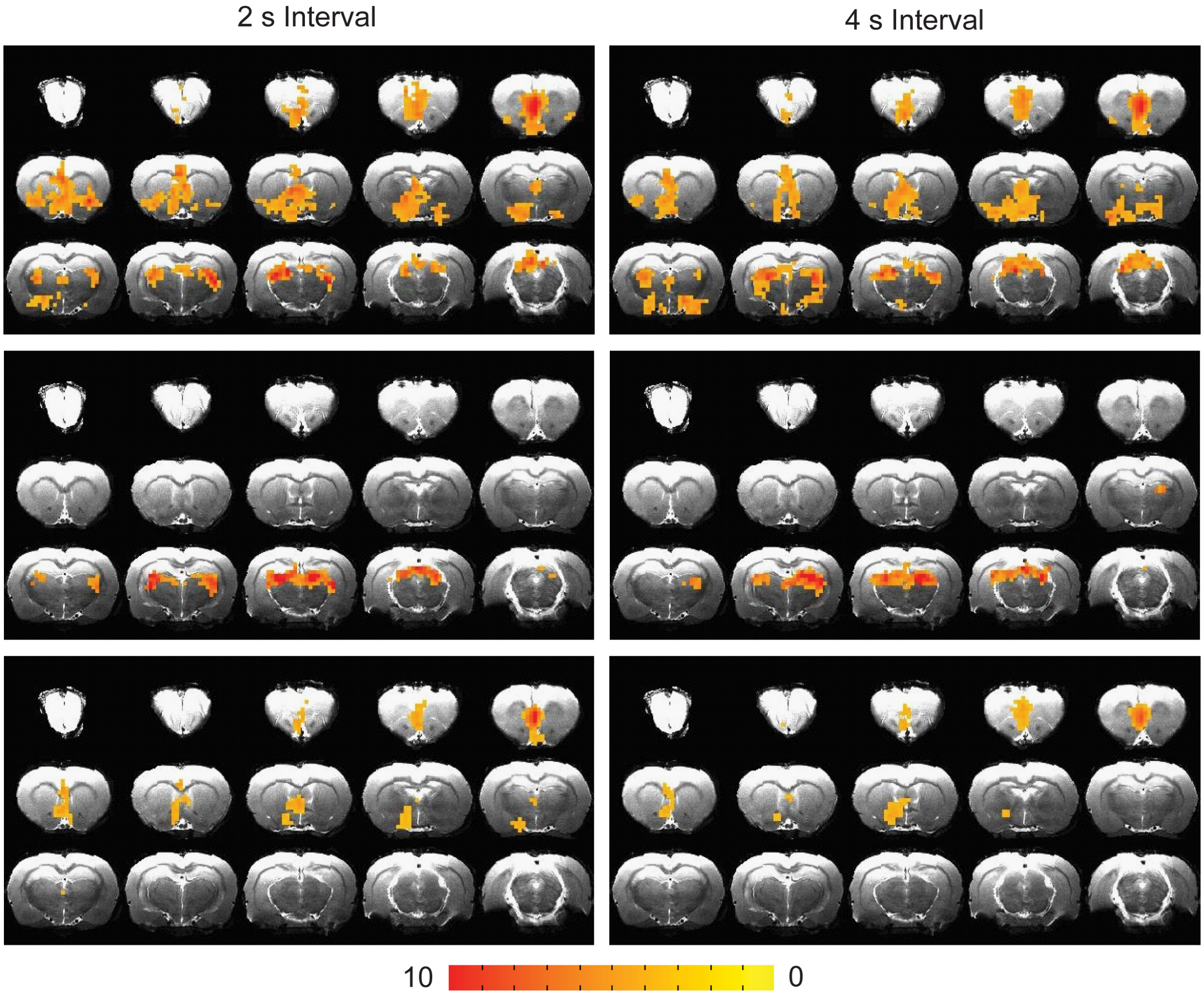

Suppl. Fig. 5. fMRI: mPFC stimulation with 2 or 4-s interval increases less BOLD signals throughout the brain. The photostimulation with the 2- or 4-s interval increased BOLD signals in extensive subcortical regions of the mPFC with ChR2 (top; n = 10), while BOLD signals in the control group were confined in the visual thalamus and superior colliculus (middle; n = 7). The 2- or 4-s interval stimulation resulted in significant differences in BOLD signal between the ChR2 and control groups in fewer regions (bottom) than the 1-s interval stimulation (Fig. 3e, bottom).

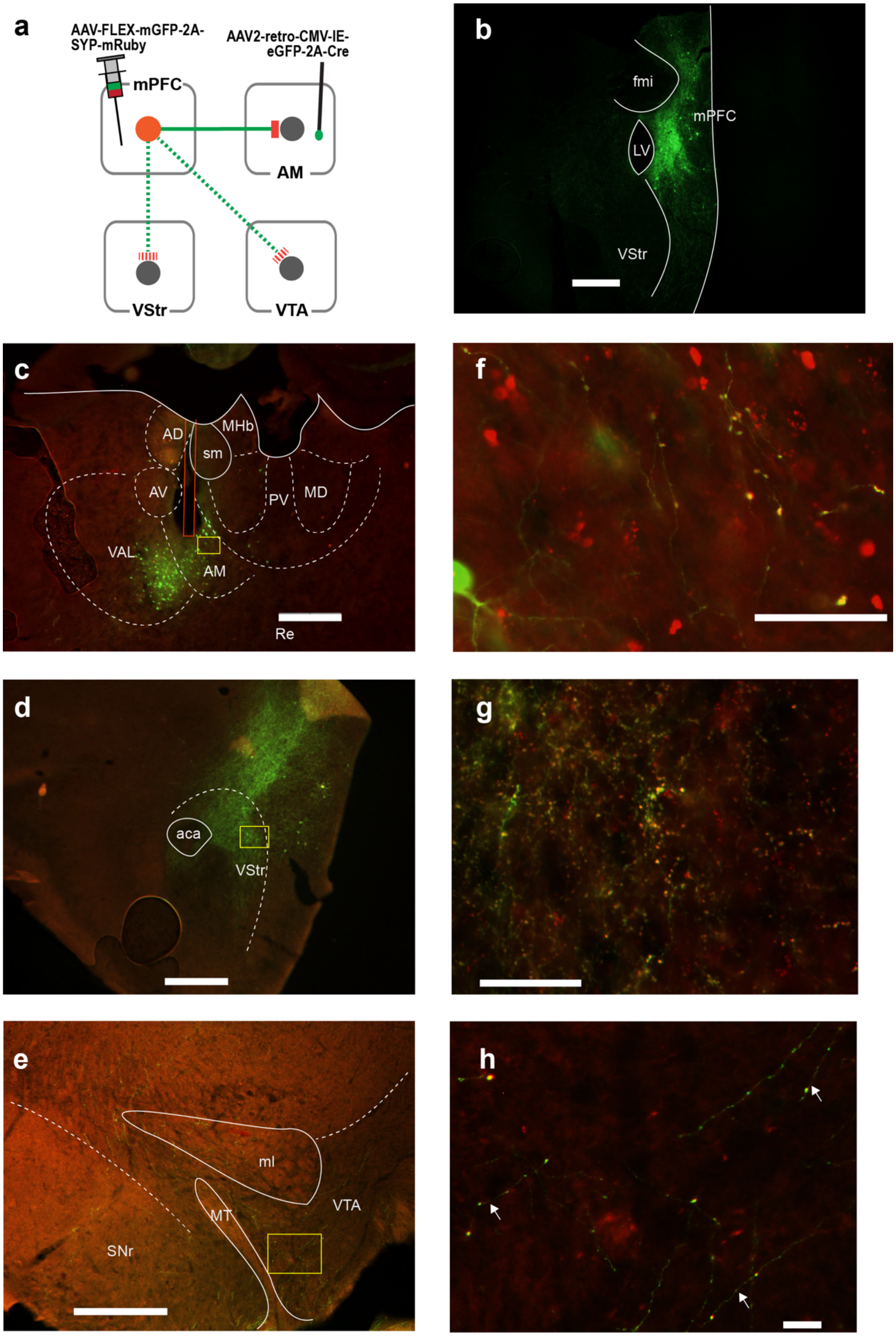

Suppl. Fig. 6. mPFC neurons projecting to the AM have collateral projections to the VStr and VTA (mouse 268). See the Fig. 5 legend. The arrows (h) indicate collateral branches with mRuby-labeled boutons. In addition to the AM, GFP-labeled cells were found in the VAL, which does not project to the mPFC [7]. Scale bar (b – e) = 250 µm; scale bar (f – h) = 25 µm.

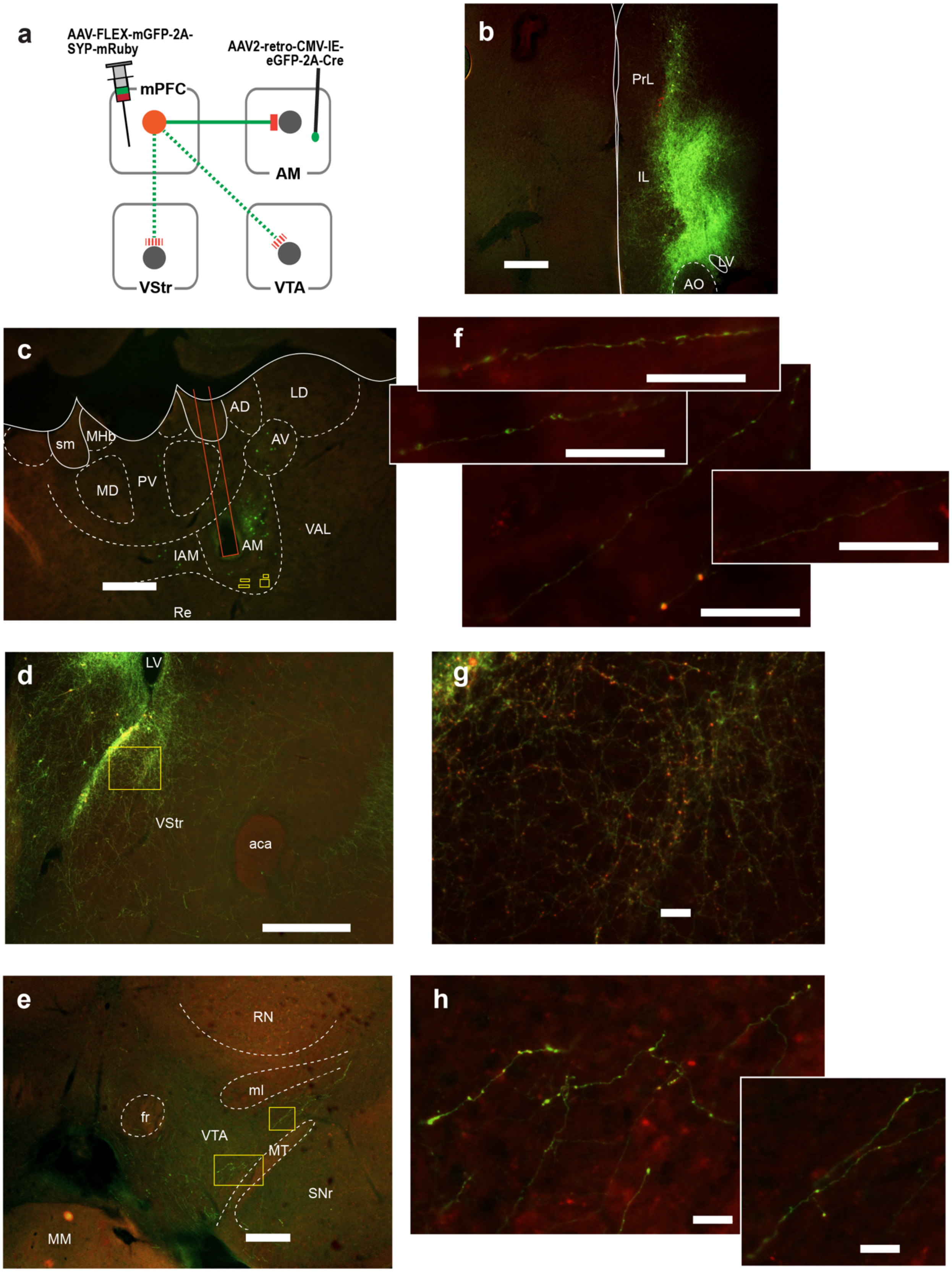

Suppl. Fig. 7. mPFC neurons projecting to the AM have collateral projections to the VStr and VTA (mouse 283). See the Fig. 5 legend. MM, medial mammillary nucleus. Scale bar (b – e) = 250 µm; scale bar (f – h) = 25 µm.

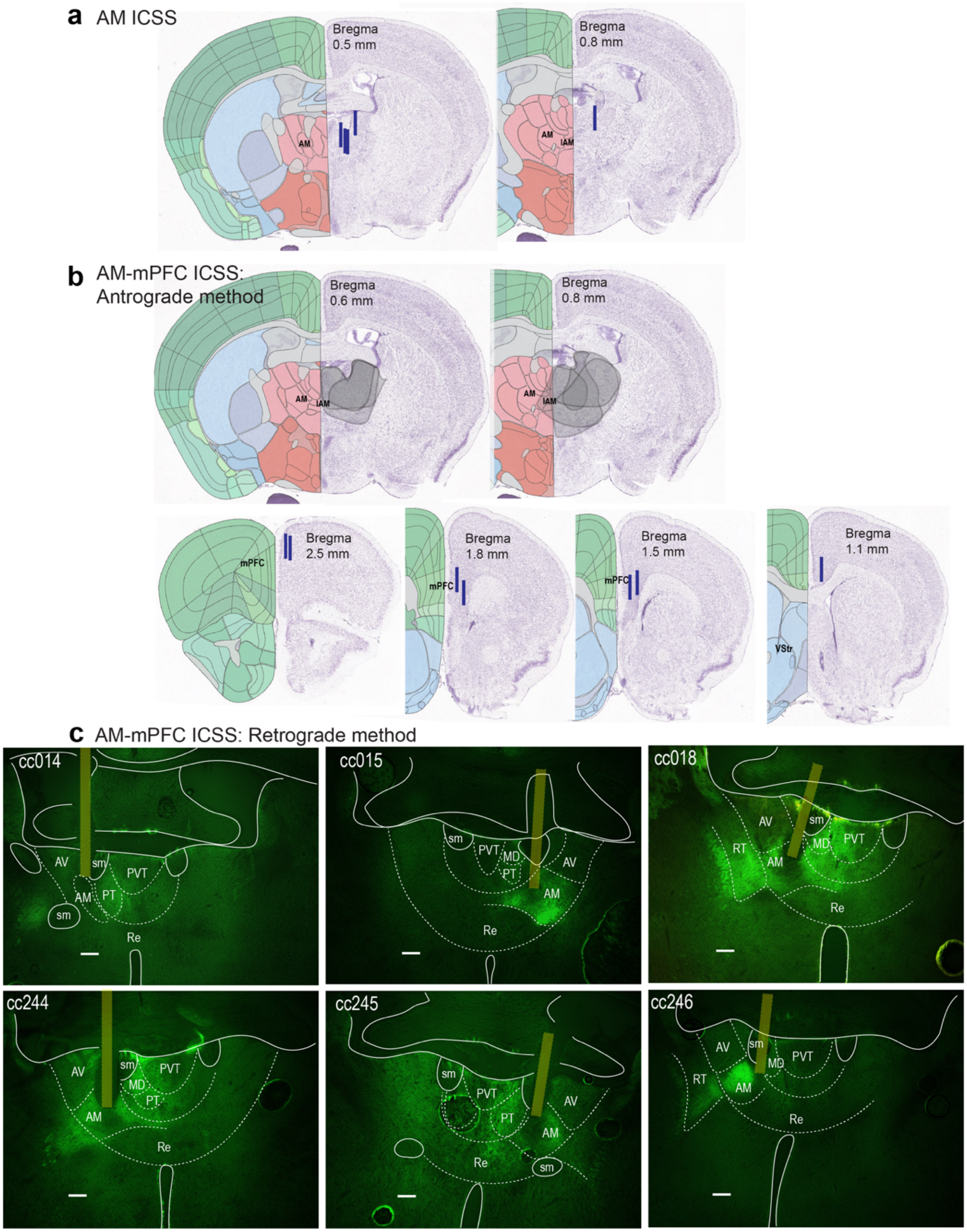

Suppl. Fig. 8. Sites showing AAV expressions and optic-fiber placements for AM experiments. Coronal sections were adopted from Allen Mouse Brain Atlas© of Allen Institute for Brain Science available at: https://mouse.brain-map.org/static/atlas. **a** Each line indicates the optic-fiber placement of each mouse used in Fig. 6a. **b** each gray area shows fluorophore-expression for each mouse (top). Each line indicates the optic-fiber placement of each mouse used in Fig. 6b (bottom). **c** Photomicrograms showing fluorophore-expressions and optic-fiber placements of 6 mice; that of the other mouse is shown in Fig. 6C (i.e., total n = 7 mice). Scale bar = 250 μm.

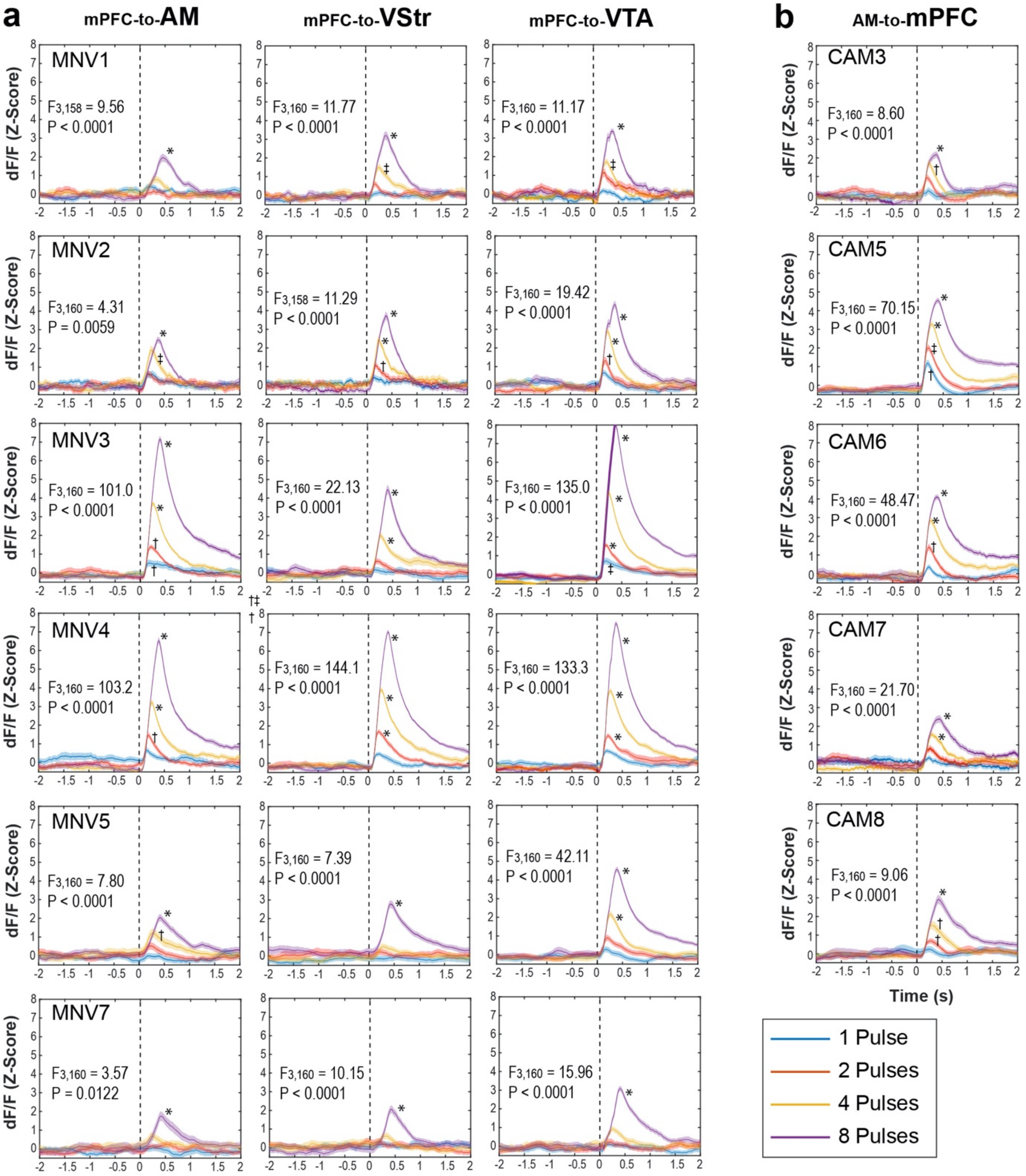

Suppl. Fig. 9. Data of individual mouse data showing that the stimulation of mPFC axonal terminals at the AM, VStr, or VTA, or of AM axonal terminals at the mPFC activates VTA DA neurons. GCaMP signals (Z-score dF/F) of VTA DA neurons induced by pulse trains (1, 2, 4 and 8) delivered at the mPFC-to-AM, mPFC-to-VStr and mPFC-to-VTA pathways (**a**) and at the AM-to-mPFC pathway (**b**). The data are means ± SEMs. The F- and P-values shown within each graph indicate the interaction with a 2^{Time} X 4^{Pulse} within-subjects ANOVA with Time (2-s block before and after stimulation train) and Pulse (1, 2, 4, and 8) on areas under curve for each region of each mouse. *$P < 0.0001$; ‡$P < 0.001$; †$P < 0.05$, paired, two-tailed t-tests with Benjamini and Hochberg correction, comparing data between the 2-s period prior and the 2-s period after the onset of photostimulation (the dotted line).

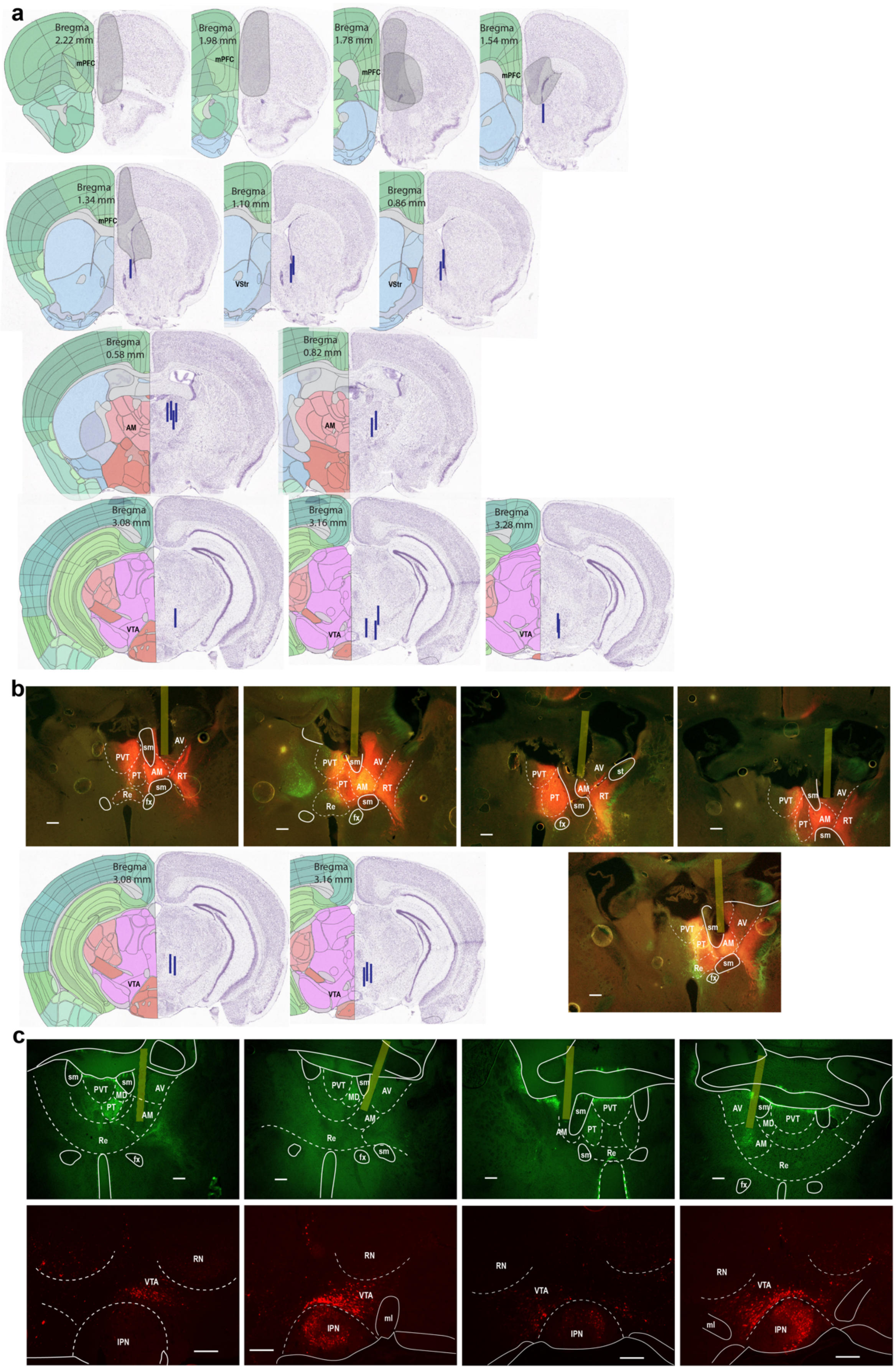
a
Bregma 2.22 mm
mPFC
Bregma 1.98 mm
mPFC
Bregma 1.78 mm
mPFC
Bregma 1.54 mm
mPFC
Bregma 1.34 mm
mPFC
Bregma 1.10 mm
VStr
Bregma 0.86 mm
VStr
Bregma 0.58 mm
AM
Bregma 0.82 mm
AM
Bregma 3.08 mm
VTA
Bregma 3.16 mm
VTA
Bregma 3.28 mm
VTA
b
PVT
sm
AV
PT
AM
RT
Re
fx
Bregma 3.08 mm
VTA
Bregma 3.16 mm
VTA
c
MD
st
RN
IPN
ml

Suppl. Fig. 10. Sites showing the expression of Chrimson and stimulation and recording sites. a-b Each gray area indicates fluorophore-expression for each mouse, and blue lines indicate optic-fiber placements for the stimulation or recording sites for the VStr, AM, and VTA. **a** Results of the mice used for the stimulation of mPFC neuron terminals at the AM, VStr and VTA. **b** Results of the mice (n = 5) used for AM-to-mPFC stimulation. Photomicrograms showing optic-fiber placements. Coronal drawing sections were adopted from Allen Mouse Brain Atlas© of Allen Institute for Brain Science available at: https://mouse.brain-map.org/static/atlas. **c** Photomicrograms showing optic-fiber placements (upper raw) and hM4D expressions indicated by mCherry (lower raw) used for the ICSS experiment (n = 4). Scale bar = 250 µm.

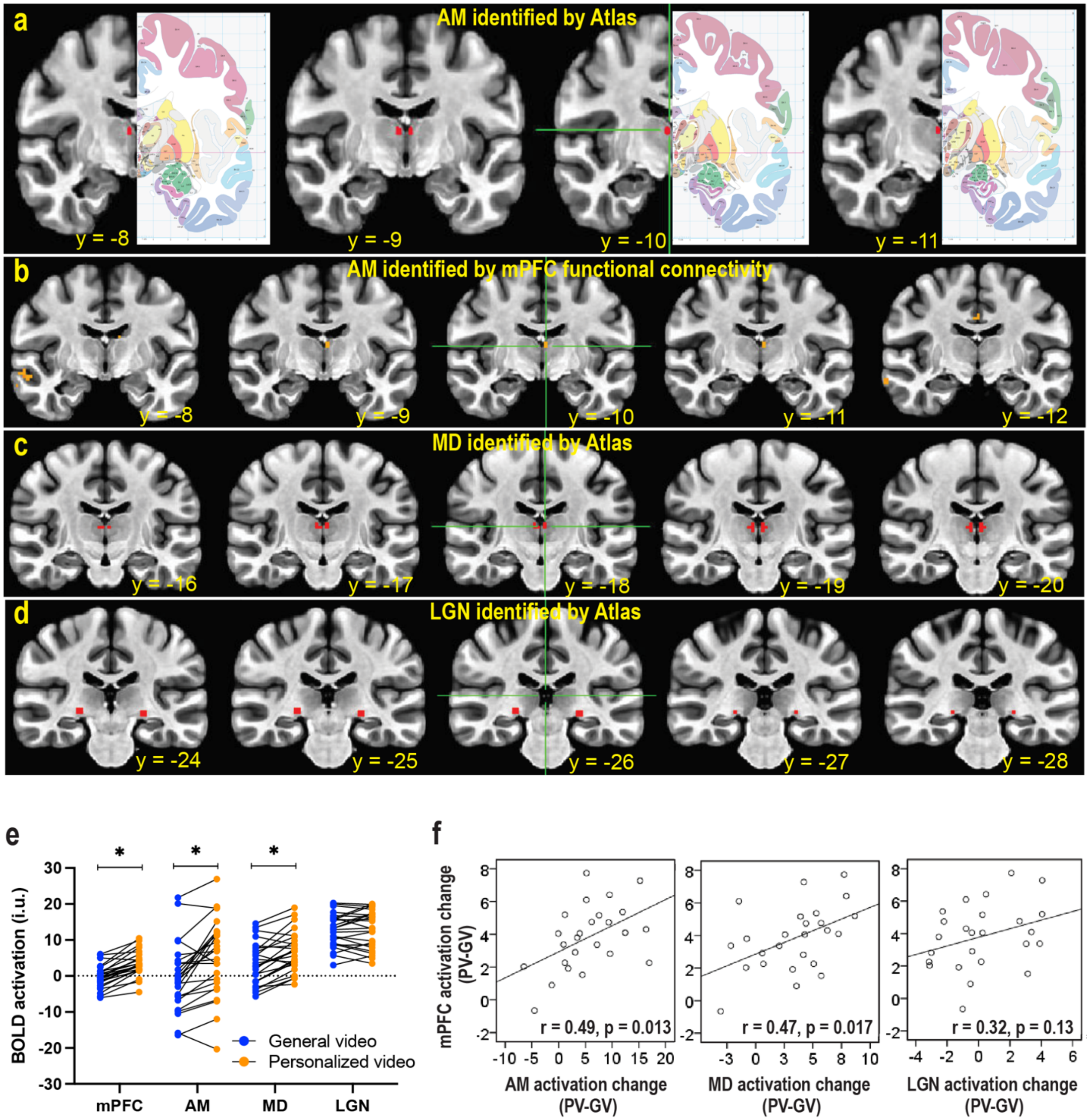

Suppl. Fig. 11. Relative locations of thalamic nuclei, functional connectivity, and their activation changes during video viewing. **a** The anteromedial thalamic (AM, red) RIO was drawn by hand in the MNI_T1_152_2009_template with reference to *Atlas of The Human Brain*[8]. **b** The AM region shows the highest thalamic functional connectivity with the medial prefrontal cortex (mPFC), indicating its functional distinction from other thalamic nuclei. **c** The mediodorsal thalamic nucleus (MD) is hand-drawn based on the atlas. **d** The later geniculate nuclei (LGN) is hand-drawn based on the atlas. **e** Brain activation in the regions of interest derived with BOLD fMRI data without spatial smoothing, to maximize the analytic capability of delineating the functional distinctions among thalamic nucleus from a spatially compacted complex. While the control region LGN did not show difference in brain activation between general video (GV) and personalized video (PV) conditions ($P = 0.56$), the mPFC, AM, and MD showed higher activation under the PV condition (*$P < 0.001$; paired, two-tailed t-test). The mPFC and AM showed selective activation in response to only PV ($P = 0.06$), but not to GV ($P = 0.57$). On the other hand, the MD responded to both PV ($P < 0.001$) and GV

conditions ($P = 0.016$). These results suggest a functional difference between AM and MD. **f** Relationships between activation changes in the thalamic nucleus and that in the mPFC are examined. Significant positive correlations are found between mPFC and AM and between mPFC and MD, but not between mPFC and LGN. When controlling for the influences of MD and LGN activations, the partial correlation between mPFC and AM is significant (partial $r = 0.48$; $P = 0.026$). In contrast, when controlling for the influences of AM and LGN activation, the partial correlation between mPFC and MD activation is not significant ($r = 0.16$, $P = 0.50$). These analyses suggest a stronger relationship between the mPFC and AM with respect to viewing personalized video contents. The similarity in activation patterns of AM to that of mPFC and the superior resting-state functional connectivity between the two regions further support the conjuncture that the mPFC-AM form a positive feedback loop involving in motivational behaviors. All p-values were obtained with paired, two-tailed t-tests.

SUPPLEMENTARY TABLES

Supplementary Table 1. List of AAVs

AAV	Titer (vg/m)	Source
AAV1-hSyn-ChR2(H134R)-EYFP	7×10^{12}	UNC
AAV1-hSyn-eNpHR3.0-EYFP	1×10^{13}	UNC
AAV1-hSyn-EYFP	3.7×10^{12}	GEVVC
AAV1-Ef1a-DIO-hChR2(H134R)-EYFP	7×10^{12}	Addgene
AAV1-Syn-ChrimsonR-tdTomato-WPRE	2.3×10^{13}	Addgene
AAV2-retro-EF1a-DIO-hChR2(H134R)-EYFP	3.5×10^{12}	GEVVC
AAV2-retro-CAG-tdTomato	7.2×10^{12}	Addgene
AAV1-Syn-FLEX-GCaMP6f-WPRE	5×10^{12} – 9×10^{12}	Addgene
AAV5-Syn-FLEX-rc[ChrimsonR-tdTomato]	2.0×10^{13}	Addgene
AAV2-retro-CMV-IE-eGFP-2A-iCre	4.1×10^{12}	GEVVC
AAV1-syn1-FLEX-mGFP-2A-SYP-mRuby	6.9×10^{11}	GEVVC
AAV-retro-hSyn-hChR2(H134R)-EYFP	1.9×10^{13}	Addgene
AAV9-hSyn-DIO-hM4D(Gi)-mCherry	2.2×10^{13}	Addgene
AAV2-hSyn-hChR2(H134R)-EYFP-WPRE-PA	5.6×10^{12}	Addgene

Supplementary Table 2. Stereotaxic coordinates for AAV injection sites.

	Region	Anterior-Posterior	Medial-Lateral	Dorsal-Ventral
Cortex	Infralimbic	+1.8	+0.4	-3.3
	Prelimbic	+1.7 – -1.8	+0.4	-1.8 – -2.1
	Medial orbital	+2.3	+0.4	-2.7
	Ventral/Lateral orbital	+2.3	+0.8 – +1.5	-2.7
	Cg1/M2	+1.8	+0.3 – +0.9	-1.0 – -1.4
	Anterior insular	+1.8	+2.0 – +2.3	-2.8 – -3.3
	Tenia tecta	+2.2	+0.3 – +0.4	-3.5 – -4.6
Septum	Septum	+1.1	+0.3	-3.5
Striatum	Ventral striatum	+1.1 – +1.3	+0.5 – +1.2	-4.2 – -4.9
	Medial dorsal striatum	+1.1	+0.9	-3.5
	Internal capsule	-0.1	+1.25	-3.4
Thalamus	Anteromedial n.	-0.8	+0.7	-3.8
	Mediodorsal n.	-1.1	+0.3	-2.7
	Reunions n.	-0.8	+0.2	-4.0
Hypothalamus	Hypothalamus	-0.6 – -2.0	+0.5 – +0.8	-4.5
Midbrain	Ventral tegmental area	-3.3	+0.8	-4.5

SUPPLEMENTARY REFERENCES

1 Öngür, D. & Price, J. L. The organization of networks within the orbital and medial prefrontal cortex of rats, monkeys and humans. *Cerebral Cortex* **10**, 206-219 (2000).

2 Uylings, H. B. M., Groenewegen, H. J. & Kolb, B. Do rats have a prefrontal cortex? *Behavioural Brain Research* **146**, 3-17 (2003).

3 Paxinos, G. & Watson, C. *The Rat Brain in Stereotaxic Coordinates*. 6th edn, (2007).

4 Franklin, K. B. J. & Paxinos, G. *The Mouse Brain in Stereotaxic Coordinates*. 3rd edn, (Elsevier, 2007).

5 Swanson, L. W. *Brain Maps: Structure of the Rat Brain*. (1992).

6 Ikemoto, S., Yang, C. & Tan, A. Basal ganglia circuit loops, dopamine and motivation: A review and enquiry. *Behav Brain Res* **290**, 17-31, doi:10.1016/j.bbr.2015.04.018 (2015).

7 Hoover, W. B. & Vertes, R. P. Anatomical analysis of afferent projections to the medial prefrontal cortex in the rat. *Brain Struct Funct* **212**, 149-179, doi:10.1007/s00429-007-0150-4 (2007).

8 Mai, J. K., Majtanik, M. & Paxinos, G. *Atlas of the human brain*. 4th edn, 456 (Academic Press, 2015).
