# Supplementary material for: Medial prefrontal cortex and anteromedial thalamus interaction regulates goal-directed behavior and dopaminergic neuron activity
Source: Nat Commun. 2022 Mar 16;13:1386. doi: 10.1038/s41467-022-28892-7 (PMC8927595; doi:10.1038/s41467-022-28892-7)
Supplement: Supplementary file 3 — Reporting summary [file 41467_2022_28892_MOESM3_ESM.pdf]

nature research

Corresponding author(s): Satoshi Ikemoto

Last updated by author(s): Dec 20, 2021

Reporting Summary

Nature Research wishes to improve the reproducibility of the work that we publish. This form provides structure for consistency and transparency in reporting. For further information on Nature Research policies, see our Editorial Policies and the Editorial Policy Checklist.

Statistics

For all statistical analyses, confirm that the following items are present in the figure legend, table legend, main text, or Methods section.

n/a	Confirmed	
☐	☒	The exact sample size (*n*) for each experimental group/condition, given as a discrete number and unit of measurement
☐	☒	A statement on whether measurements were taken from distinct samples or whether the same sample was measured repeatedly
☐	☒	The statistical test(s) used AND whether they are one- or two-sided<br>*Only common tests should be described solely by name; describe more complex techniques in the Methods section.*
☐	☒	A description of all covariates tested
☐	☒	A description of any assumptions or corrections, such as tests of normality and adjustment for multiple comparisons
☐	☒	A full description of the statistical parameters including central tendency (e.g. means) or other basic estimates (e.g. regression coefficient) AND variation (e.g. standard deviation) or associated estimates of uncertainty (e.g. confidence intervals)
☐	☒	For null hypothesis testing, the test statistic (e.g. *F*, *t*, *r*) with confidence intervals, effect sizes, degrees of freedom and *P* value noted<br>*Give P values as exact values whenever suitable.*
☐	☒	For Bayesian analysis, information on the choice of priors and Markov chain Monte Carlo settings
☒	☐	For hierarchical and complex designs, identification of the appropriate level for tests and full reporting of outcomes
☒	☐	Estimates of effect sizes (e.g. Cohen's *d*, Pearson's *r*), indicating how they were calculated

Our web collection on statistics for biologists contains articles on many of the points above.

Software and code

Policy information about availability of computer code

Data collection	ICSS behavior was performed in the operant conditioning chambers, controlled by Med-PC IV v4.39.<br>Video-recorded behaviors were quantified with the video tracking software Noldus EthoVision XT v15.<br>c-Fos-ir cells were detected with ImageJ v1.51 (NIH).<br>Fiber-photometry calcium signals were recorded with a Fiber Photometry System (Doric Lenses) with the Doric Neuroscience Studio software v4.<br>Magnetic resonance imaging (MRI) data for human experiment were collected using a Siemens 3.0-T scanner (MAGNETOM Prisma, Siemens Healthcare Erlangen, Germany). The presentation of fMRI task stimuli was controlled by the software E-prime 3.0 (https://pstnet.com/products/e-prime/).<br>MRI data for rat experiment were acquired on a Bruker Biospin 9.4T scanner (Bruker Medizintechnik, Karlsruhe, Germany) using a surface circular coil and birdcage volume transmit coil.

Data analysis	Fiber photometry data were processed with house-written MATLAB (R2016b) code [https://zenodo.org/badge/latestdoi/337120575] to transform raw photometry recordings into a dF/F, extract the area under the curve (AUC) before and after optogenetic stimulations.<br>GraphPad Prism v4 & v8 or Statistica v6.1 were used for all statistical comparisons except for MRI data.<br>Electrophysiological recording data were analyzed with Molecular Devices' Clampfit v10.6 and Origin Pro v9.2 (OriginLab Corporation, MA, USA).<br>MRI data were processed using open source software AFNI (ver: AFNI_18.3.10, https://afni.nimh.nih.gov/), ANTs (ver: 2.2.0, http://stnava.github.io/ANTs/), and SPM12 (ver: v7771, https://www.fil.ion.ucl.ac.uk/spm). While AFNI and ANTs were standalone, SPM was implemented on Matlab (ver:2017b, The Math Works Inc, https://www.mathworks.com). No custom algorithms or software were developed for this research. ROI level correlations between behavioral and imaging measures as well as descriptive statistical analysis on demographic information such as age mean and SD were performed using SPSS (ver: 22, https://www.ibm.com/analytics/spss-statistics-software).

For manuscripts utilizing custom algorithms or software that are central to the research but not yet described in published literature, software must be made available to editors and reviewers. We strongly encourage code deposition in a community repository (e.g. GitHub). See the Nature Research guidelines for submitting code & software for further information.

Data

Policy information about availability of data

All manuscripts must include a data availability statement. This statement should provide the following information, where applicable:

- Accession codes, unique identifiers, or web links for publicly available datasets
- A list of figures that have associated raw data
- A description of any restrictions on data availability

Data availability statement is included in the paper.

Field-specific reporting

Please select the one below that is the best fit for your research. If you are not sure, read the appropriate sections before making your selection.

☒ Life sciences ☐ Behavioural & social sciences ☐ Ecological, evolutionary & environmental sciences

For a reference copy of the document with all sections, see nature.com/documents/nr-reporting-summary-flat.pdf

Life sciences study design

All studies must disclose on these points even when the disclosure is negative.

Sample size	Our sample size was determined by the variability of acquired data and the magnitude of manipulation effect (i.e., signal-to-noise ratio). When the hypothesis is to show a difference between manipulation conditions and when statistical analysis confirms the difference, then we assumed that the sample size must have been large enough. Based on this premise, we estimate minimal sample sizes for our physiological and behavioral experiments in animals are n = 4. The fMRI experiments in rats and humans used the minimal sizes of n = 7 rats and n = 23 humans. For neural connectivity experiments, we assumed that animals raised in the same normal laboratory condition must have essentially the same neural connectivity. When we observed essentially the same results in n = 3 animals, we suggested reliable connections between structures.
Data exclusions	Data were excluded from analyses when histological results did not verify the brain regions intended for manipulation or recording. Such experiments include those described in Figs. 3b, 6a-6e, 7a-7g, and Suppl. Figs. 3b-3f. fMRI data were excluded when significant head motions were detected for the data described in Fig. 8 and Suppl. Fig. 11.
Replication	All experiments we performed are reported in the paper. However, some original experiments are not reported because of the following reasons: (1) Fig. 5 and Suppl. Figs. 6-7 show data that replicated original experiments (n = 2), which were not shown in the paper due to two reasons: extended diffusions of infused AAV and poor photomicrogram quality. (2) The data described in Fig. 6c were a replication of original experiment in which intra-AM AAV injections appeared to have produced toxic effects in infected cells; the original data are not shown in the paper. (3) The data described in Fig. 6d were a replication of original experiment in which retrograde expressions of AAV were observed and appeared to have interfered with the effects of anterograde expressions of AAV.
Randomization	For between-subjects designed experiments, animals were randomly assigned to experimental groups. For within-subjects designed experiments, including Human and rat fMRI experiments, experimental conditions were delivered in a counterbalanced manner.
Blinding	The animal experiments were performed without any blinding procedure. All behavioral data were automatically collected in isolated chambers with electronic devices, which executed programed conditions in the absence of the experimenter.<br>Similarly, human fMRI data were automatically collected by the scanner during which the experimenter did not interact with the participants. In addition, electronic devices delivered experimental video clips and control video clips in a counterbalanced order (i.e., a within-subjects design).

Reporting for specific materials, systems and methods

We require information from authors about some types of materials, experimental systems and methods used in many studies. Here, indicate whether each material, system or method listed is relevant to your study. If you are not sure if a list item applies to your research, read the appropriate section before selecting a response.

Materials & experimental systems

n/a	Involved in the study
x	Antibodies
x	Eukaryotic cell lines
x	Palaeontology and archaeology
	x Animals and other organisms
	x Human research participants
x	Clinical data
x	Dual use research of concern

Methods

n/a	Involved in the study
x	ChIP-seq
x	Flow cytometry
	x MRI-based neuroimaging

Animals and other organisms

Policy information about studies involving animals; ARRIVE guidelines recommended for reporting animal research

Laboratory animals	The study used male and female mice and male rats with 2.5 - 6 months of age. All mice and rats were individually housed in a vivarium and maintained at consistent temperature (70–74 °F) and humidity (35–55%).
Wild animals	The study did not involve wild animals.
Field-collected samples	The study did not involve samples collected from the field.
Ethics oversight	All procedures were approved by the Animal Care and Use Committee of the Intramural Research Program of National Institute of Drug Abuse and were in accordance with the National Research Council Guide for the Care and Use of Laboratory Animals.

Note that full information on the approval of the study protocol must also be provided in the manuscript.

Human research participants

Policy information about studies involving human research participants

Population characteristics	Twenty-five healthy students (M = 11; F = 14) participated in this study (age: mean (std.) = 24.1 (2.48), range = 21 and 30 yr). All the participants had normal or corrected-to-normal vision and reported no neurological diseases.
Recruitment	Subjects were recruited by advertisements through Internet media including forums and social medial platforms. This is a typical and standard procedure to recruit participants and is not expected to produce experimenter's bias in recruitment to influence fMRI results in one way or another.
Ethics oversight	This study was approved by the local ethical committee of Zhejiang University. Written informed consents were obtained from every participant before experiment.

Note that full information on the approval of the study protocol must also be provided in the manuscript.

Magnetic resonance imaging

Experimental design

Design type	Human fMRI experiment: Block designed task fMRI, resting-state fMRI.<br>Rat fMRI experiment: Block designed optogenetic stimulus fMRI (opto-fMRI).
Design specifications	Human task fMRI: The experiment adopted a block design with IVs and CVs, each type of clips was presented in 6 1-min blocks. The two types of the 6 blocks were presented alternatively and separated by a 30-s break, during which the participants viewed a white fixation on a black background. The order of VIs and CVs was counterbalanced between subjects.<br><br>Rat opto-fMRI: Block-design optogenetic stimulation was delivered to the right mPFC under three conditions: 25-Hz trains with an interval of 1s, 2s, or 4s, respectively. Each condition consisted of 5 blocks, and each block consisted of 20s stimulus on and 40s off. Two scan sessions with the stimulus order of A) 1s-2s-4s-interval or B) 4s-2s-1s-interval were performed. The order of scans A and B was counterbalanced between animals.
Behavioral performance measures	Human task fMRI: this is a passive video watching task. To minimize disruption of viewing state, no additional question or interruption was introduced during the experimental session. Questionnaires were administrated to ask how the subjects like the videos.<br>Rat fMRI: During the scanning, animals were kept anesthetized with a combination of isoflurane (0.5%) and dexmedetomidine hydrochloride (0.015 mg/kg/hr). No behavioral measures were taken. Heart rate and blood oxygenation levels were continuously monitored using a noninvasive pulse oximetry attached to the animal's hind foot, while respiration rate was monitored with a MouseOx sensor (Starr Life Sciences, Oakmont, PA, USA) beneath the animal's chest. Respiration rate, oxygenation and heart rate varied between 65 to 80 cycles/min, 90% - 100% and 250-320 BMP, respectively, during functional MRI (fMRI) data acquisition.

Acquisition

Imaging type(s)
Functional and structural images

Field strength
3T for humans and 9.4T for rats

Sequence & imaging parameters
Human structural MRI: High resolution anatomical images were acquired using a T1-weighted magnetization prepared rapid gradient echo sequence with parameters below: TR = 2300 ms, TE = 2.32 ms, voxel size = 0.90 × 0.90 × 0.90 mm3, flip angle = 8°, field of view = 240 mm2, voxel matrix = 256 × 256.

Human fMRI: FMRI data were collected using a T2*-weighted gradient echo planar imaging sequence with multi-bands acceleration (TR = 1000 ms, TE = 34 ms, slice thickness = 2.50 mm, voxel size = 2.50 × 2.50 × 2.50 mm3, voxel matrix = 92 × 92, flip angle = 50 °, field of view = 230 mm2, slices number = 52, MB-factor = 4).

Rat structural MRI: High-resolution anatomical images were acquired using a Rapid Acquisition with Relaxation Enhancement (RARE) sequence (repetition time (TR) = 2200 ms, FOV = 35×35 mm2, slice thickness = 0.5 mm, slice number = 30).

Rat fMRI: fMRI data were acquired using a T2*-weighted EPI sequence (TE = 13 ms, TR = 1000 ms, segment = 2, FOV = 35 × 35 mm2, matrix size = 64×64, slice thickness = 1 mm, slice number = 15).

Area of acquisition
Whole brain for both humans and rats

Diffusion MRI ☐ Used ☒ Not used

Preprocessing

Preprocessing software
Human fMRI: Preprocessing of fMRI data included the following steps. First, slice time correction and head motion correction were performed using AFNI (https://afni.nimh.nih.gov/). Then, tissue segmentation was then conducted to extract brains using SPM12 (https://www.fil.ion.ucl.ac.uk/spm/). Structural and functional images were co-registered and normalized into the MNI space using ANTs (http://stnava.github.io/ANTs/). Finally, spatial smoothing was applied to the normalized fMRI data with a 5 mm full-width-at-half-maximum Gaussian kernel. For resting-state data, two more preprocessing steps were included: 1) nuisance variable regression including six-rigid head motion and their forward derivates, fame-displacement (FD), and the first 5 principle components from white matter and cerebral spinal fluid (CSF) separately; and 2) a band-pass filtering (0.01Hz – 0.1Hz) was applied.

Rat fMRI: FMRI data were preprocessed with slice timing correction, head motion correction, spatial normalization and smoothing (full-width-at-half-maximum 1.25 mm). Spatial normalization was performed using ANTs (http://stnava.github.io/ANTs/).

Normalization
For both human and rat data, a two-step strategy was used to optimize the spatial normalization. High resolution structural images were used to estimate both linear (12 degree) and nonlinear transformation parameters from anatomic space to the template. Linear transformation parameters (6 degrees for rigid transformation) were estimated to align individual's fMRI data (the mean image after head motion correction) to corresponding structural images. Then normalization to template space from native fMRI space was achieved by combining these three sets of transformations 1) fMRI-to-structure rigid matrix, 2) structure-to-tempate affine matrix, and 3) structure-to-template nonlinear parameters.

Normalization template
ICBM152 was used for human MRI normalization;
A previously published rat template (Lu et al., 2012) was used for rat MRI normalization.

Noise and artifact removal
For human task fMRI and rat opto-fMRI data, 6 motion parameters were included in the first level analysis to remove motion induced artifact.

For human resting-state fMRI data: 1) nuisance variable regression including six-rigid head motion and their forward derivates, famewise displacement (FD), and the first 5 principle components from white matter and cerebral spinal fluid (CSF) separately; and 2) a band-pass filtering (0.01Hz – 0.1Hz) was applied. No global regression, no ICA was used.

Volume censoring
Censoring was performed in human fMRI data only. Follow a previous study by Power and colleagues, framewise displacement (FD, Eq. 1) of fMRI data was calculated for each participant as indices of head motion for task and resting-state data separately.
Eq. 1: $FD_i = |\Delta d_{ix}| + |\Delta d_{iy}| + |\Delta d_{iz}| + |\Delta \alpha_i| + |\Delta \beta_i| + |\Delta \gamma_i|$
where $\Delta d_{ix} = d_{(i-1)x} - d_{ix}$, and similarly for the other rigid body parameters [d_{ix} d_{iy} d_{iz} α_i β_i γ_i].
If FD >0.5mm, the two time point that producing FD were censored. A participant would be excluded from statistical analysis if her/his mean FD >0.3mm, or the total number of frames censored with FD > 0.5mm is more that 10% of the total length of the data. With these two criteria, two subjects were excluded from task fMRI data analysis, and one subject was excluded from resting-state fMRI statistical analysis.

Censoring was not performed for rat fMRI data as the animals were kept anesthetized during fMRI scanning.

Statistical modeling & inference

Model type and settings	For both human task and rat optogenetic fMRI data, the first level analysis was conducted using general linear modelling (GLM) implemented with AFNI's command 3Ddeconvovle. Drift and auto-correlation were modelled in the program with default settings. Censoring was used in human fMRI data analysis only.
Effect(s) tested	Paired t-tests were conducted to compare brain activation difference between task condition (VIs vs CVs) in human fMRI statistical analysis using 3dttest++; Independent two sample t-tests were conducted to compare brain activation difference between two groups of rats received AAV1-hSyn-ChR2(H134R)-EYFP (n=10) or AAV1-hSyn-EYFP (n=7). Correlations between brain activation and self-stimulation behavior (lever press) were examined using Pearson's correlation coefficients.

Specify type of analysis: ☐ Whole brain ☐ ROI-based ☒ Both

Anatomical location(s)	Anterior medial (AM) thalamus in human fMRI was determined and hand-drawn on the MNI_T1_152_2009_template with reference to the 'Atlas of The Human Brain' (Mai JK, Majtanik M, Paxinos G. Atlas of the human brain, 4th edMai JK, Majtanik M, Paxinos G. Atlas of the human brain, 4th edn. Academic Press (2015)).<br>The mPFC region was defined using task activation and resting state functional connectivity.

Statistic type for inference<br>(See Eklund et al. 2016)	Independent two-sample t-test, one sample t-tests, paired wise t-test was conducted based on questions to answer.<br><br>Human voxel wise fMRI results were corrected for multiple comparisons (corrected P < 0.05, Fig 8a-c) using randomization and permutation simulation in AFNI (single voxel p-value <0.001 and minimum cluster size =33 voxels).<br><br>Rar fMRI results were corrected for multiple comparisons using randomization and permutation simulation in AFNI's program 3dttest++ to achieve corrected P < 0.05, which was determined by single voxel p-value <0.005 and minimum cluster size =20 voxels.
Correction	FWE, Permutation and simulation

Models & analysis

n/a	Involved in the study
☐	☒ Functional and/or effective connectivity
☒	☐ Graph analysis
☒	☐ Multivariate modeling or predictive analysis

Functional and/or effective connectivity	Pearson correlation was calculated and transformed with Fisher's r-to-z formula to define resting state functional connectivity; Effective connectivity was estimated using Psychophysiological interactions (PPI) and dynamic causal modelling (DCM).
